# Influence of comorbidities, geriatric syndromes, and frailty on mortality risk by discharge destination in older adults after acute hospitalization: a nationwide cohort study

**DOI:** 10.3389/fpubh.2026.1754972

**Published:** 2026-02-27

**Authors:** Sunyoung Kim, Jae-ryun Lee, Kyeongeun Kim, Jungha Park, Keehyuck Lee, Hye Yeon Koo, Eunbyul Cho, Hyejin Lee

**Affiliations:** 1 Department of Family Medicine, Kyung Hee University Medical Center, Kyung Hee University College of Medicine, Seoul, Republic of Korea; 2 Center for Digital Health, Medical Science Research Institute, Kyung Hee University College of Medicine, Seoul, Republic of Korea; 3 Department of Family Medicine, Seoul National University Bundang Hospital, Seongnam, Republic of Korea; 4 Department of Family Medicine, Seoul National University, Seoul, Republic of Korea; 5 Department of Family Medicine, Hallym University Dongtan Sacred Heart Hospital, Hwaseong, Republic of Korea

**Keywords:** comorbidities, frailty, geriatric syndrome, older (diseased) population, post-discharge

## Abstract

**Background:**

This study investigated its impact of discharge destination on mortality risk among older adults following acute hospital discharge, focusing on the effects of frailty, geriatric syndromes, and comorbidities.

**Methods:**

Nationwide claims data from the South Korean National Health Insurance Service of individuals aged ≥65 years who were discharged from acute care hospitals in 2017 were retrospectively analyzed, with participants followed for mortality outcomes over 4 years. Multivariable Cox proportional hazards models were used to estimate adjusted hazard ratios (aHR) for mortality according to discharge destination and geriatric status.

**Results:**

This study included 1,115,556 participants (mean age, 75.5 years; 45.6% men). The most common discharge destination was home (76.5%), followed by tertiary/general hospitals (15.2%), long-term care hospitals (5.2%), hospitals (2.3%), and other facilities (0.8%). Patients discharged to long-term care hospitals were older, had a higher comorbidity burden, and more frequently had disabilities or geriatric syndromes than their counterparts. Mortality risk was significantly higher among those discharged to tertiary/general hospitals (aHR 1.806, 95% CI: 1.793–1.820), general hospitals (aHR 1.480, 95% CI: 1.453–1.507), and long-term care hospitals (aHR 2.922, 95% CI: 2.892–2.952) than among those discharged to home. Higher Charlson comorbidity index (≥3), more geriatric syndromes, and severe frailty were all independently associated with increased mortality risk.

**Conclusion:**

Discharge destination, frailty, geriatric syndromes, and comorbidities independently and interactively influenced the mortality risk in older adults after acute hospitalization. Tailored post-discharge management strategies are necessary, particularly for patients with frailty and multimorbidity in community settings.

## 1 Background

The global trend of an aging population has increased, and South Korea is experiencing one of the most rapid demographic transitions worldwide. As of 2025, individuals aged ≥65 years constitute >20% of the total population, and this proportion will reach 40.1% by 2050 (1). Particularly notable is the sharp increase in the population aged ≥75 years, which poses substantial challenges to the healthcare and social welfare systems (2, 3).

Population aging is reshaping healthcare utilization patterns, especially in the context of acute care hospitalization and post-discharge management (4, 5). The environment in which older adults are discharged following an acute hospital stay, whether at home, a rehabilitation hospital, or a long-term care facility, has emerged as a critical public health concern, significantly influencing both survival and quality of life (6–8). Studies based on U.S. Medicare data have demonstrated that the 1 year mortality rate for patients discharged to nursing facilities 64%, compared to 11% for those discharged to home, underscoring the crucial role of post-discharge care environments in determining patient outcomes (9). Other studies have also demonstrated significant differences in 1-year mortality, readmission rates, and quality of life according to discharge destination (home, inpatient rehabilitation facility, or nursing home) after stroke (10).

Comorbidities, geriatric syndromes, and frailty are interrelated factors that profoundly affect health outcomes in older adults (11). Comorbidities are defined as co-occurring, etiologically independent chronic health conditions and are considered important predictors of survival, poor functional status, reduced quality of life, the possibility of a higher risk of adverse events in response to medication, and greater use of healthcare services (12, 13). Frailty, characterized by increased vulnerability to stressors, such as falls, delirium, and malnutrition, is associated with more than double the 1-year mortality rate compared with its counterparts (51% vs. 25%) (14). Geriatric syndromes, including functional impairment and cognitive decline, are significant predictors of mortality, particularly when present with multiple comorbid conditions (15). The coexistence of multiple geriatric syndromes and comorbidities exponentially increases the mortality risk, highlighting the complex interplay between these factors (16). A comprehensive understanding of individual geriatric conditions and their interactions is crucial for improving prognostic outcomes and developing tailored interventions for vulnerable older adults (17).

In clinical practice, this multidimensional vulnerability most commonly manifests through the co-occurrence of multimorbidity, geriatric syndromes, and frailty, which together shape health trajectories during acute hospitalization and the post-discharge period. However, much of the existing evidence has been derived from single-center or regionally restricted cohorts and has typically examined discharge destination, frailty, comorbidity burden, or geriatric syndromes as isolated predictors (18–20). Consequently, the population-level prognostic impact of their combined and interactive effects on post-discharge mortality remains insufficiently characterized. To address this gap, we analyze nationwide data from more than 1.1 million older adults to quantify how discharge destination interacts with frailty, geriatric syndromes, and comorbidity burden in determining mortality risk after hospitalization, thereby moving beyond discharge destination alone as a proxy for risk and better capturing multidimensional vulnerability in older patients.

Hence, the present study utilized nationwide big data from South Korea to analyze the relationships among discharge destination, comorbidities, geriatric syndromes, and frailty and to investigate their effects on mortality risk. This study aimed to provide evidence to optimize post-discharge management strategies and improve outcomes for vulnerable older patients.

## 2 Methods

### 2.1 Data source and study population

This retrospective cohort study used population-based data from the National Health Insurance Service (NHIS) of South Korea. The NHIS is mandatory and covers approximately 97% of the South Korean population. The medical expenses of the remaining 3% of the population are fully covered through the government's Medical Aid programs. NHIS beneficiaries are responsible for approximately 20%−30% of their out-of-pocket medical expenses, whereas Medical Aid recipients have no out-of-pocket payments. Healthcare providers submit claims for both NHIS beneficiaries and Medical Aid recipients to the NHIS, resulting in the near-complete integration of patient healthcare information within the NHIS database. This database is a comprehensive resource that encompasses healthcare utilization patterns, diagnostic information, and prescription drug details (21). This study was approved by the Institutional Review Board of Seoul National University Bundang Hospital (X-2406-904-901). The requirement for informed consent was waived owing to the retrospective study design.

To evaluate the impact of frailty on healthcare utilization and mortality, a retrospective cohort was constructed using 4 years of NHIS data from 2017 to 2020. The year 2017 was selected as the baseline period. Among the 1,137,491 individuals aged ≥65 years who were discharged at least once from acute care hospitals (tertiary or general hospitals) between January 1 and December 31, 2017, those with missing data on key variables (*n* = 16,562), or those with unclear discharge destinations (*n* = 5,373) were excluded. Consequently, comorbidities, geriatric syndromes, and frailty were comprehensively assessed for the final study population of 1,115,556 individuals. Subsequently, mortality and healthcare utilization were tracked for 3 years (2018–2020) according to comorbidities, geriatric syndromes, and frailty status.

### 2.2 Definition of variables

Comorbidities were assessed using the Charlson comorbidity index (CCI), calculated from diagnostic information recorded during the baseline period, in accordance with the validated ICD-10 coding algorithm (Supplementary Table 1-1) (22). Geriatric syndromes were identified using claims data from the National Health Insurance Service (NHIS) database, based on predefined ICD-10 diagnostic codes. The syndromes included delirium (F05, F05.0, F05.1, F05.8, F05.9), pressure ulcers (L89), incontinence (N39.3, N39.4, F98.1, F98.5, R15, R32), and osteoporosis-related fractures (S72.0, S52.5, S22-32), consistent with previously published NHIS-based studies (23). Frailty was evaluated using the multimorbidity frailty index (mFI), a 38-item deficit accumulation measure constructed from ICD-10 diagnostic codes, following the standardized approach originally proposed and subsequently validated in large population-based studies (Supplementary Table 1-2) (24). This ICD-10–based mFI has been extensively applied and validated in analyses using the NHIS database, demonstrating good construct validity through consistent associations with mortality, healthcare utilization, and adverse health outcomes in older adults (25, 26).

Each deficit was coded as present or absent, and the mFI score was calculated as the ratio of accumulated deficits to the total number of possible deficits, yielding a continuous score ranging from 0 to 1. Participants were categorized into four frailty groups—fit, mildly frail, moderately frail, and severely frail—according to established cut-off values used in prior validation studies.

### 2.3 Outcome of interest

Discharge destinations following acute hospitalization among study participants were assessed using 2017 data. Discharge destination was defined as the facility where the patient was admitted within 1 month of discharge. If no subsequent admissions occurred, the patient was assumed to have been discharged. Admissions to facilities, such as traditional medicine clinics or public health centers, were classified as “other” discharge destinations. Discharge destinations were categorized as tertiary/general hospitals, hospitals, long-term care hospitals (LTCH), home, and other facilities.

Healthcare utilization in South Korea is categorized into emergency, outpatient (OPD), and inpatient services, serving as comprehensive indicators of overall healthcare patterns. Each service type is analyzed based on distinct claim data submitted separately, reflecting the structured approach to healthcare delivery and resource allocation. This classification facilitates systematic analysis and policy development within the Korean healthcare system (27–29).

The outcome of interest was the all-cause mortality. Participants were followed up from January 1, 2018 until the date of death or the last available date in the database (December 31, 2020), whichever occurred first.

### 2.4 Statistical analysis

Descriptive analysis was performed to summarize baseline characteristics, discharge destinations, and utilization by comorbidities, geriatric syndrome, and frailty status in 2017. Continuous variables in the text and tables are presented as means ± standard deviations (SDs), whereas categorical variables are reported as percentages.

Cox proportional hazards models were used to evaluate the association between discharge destination and all-cause mortality, stratified by comorbidity, geriatric syndrome, and frailty status, while adjusting for age, sex, income, region, and disability. Adjusted hazard ratios (aHRs) and 95% confidence intervals (CIs) were determined. All statistical analyses were conducted using the SAS software (version 9.4; SAS Institute, Cary, NC, USA).

## 3 Results

### 3.1 General characteristics

**Figure 1:**
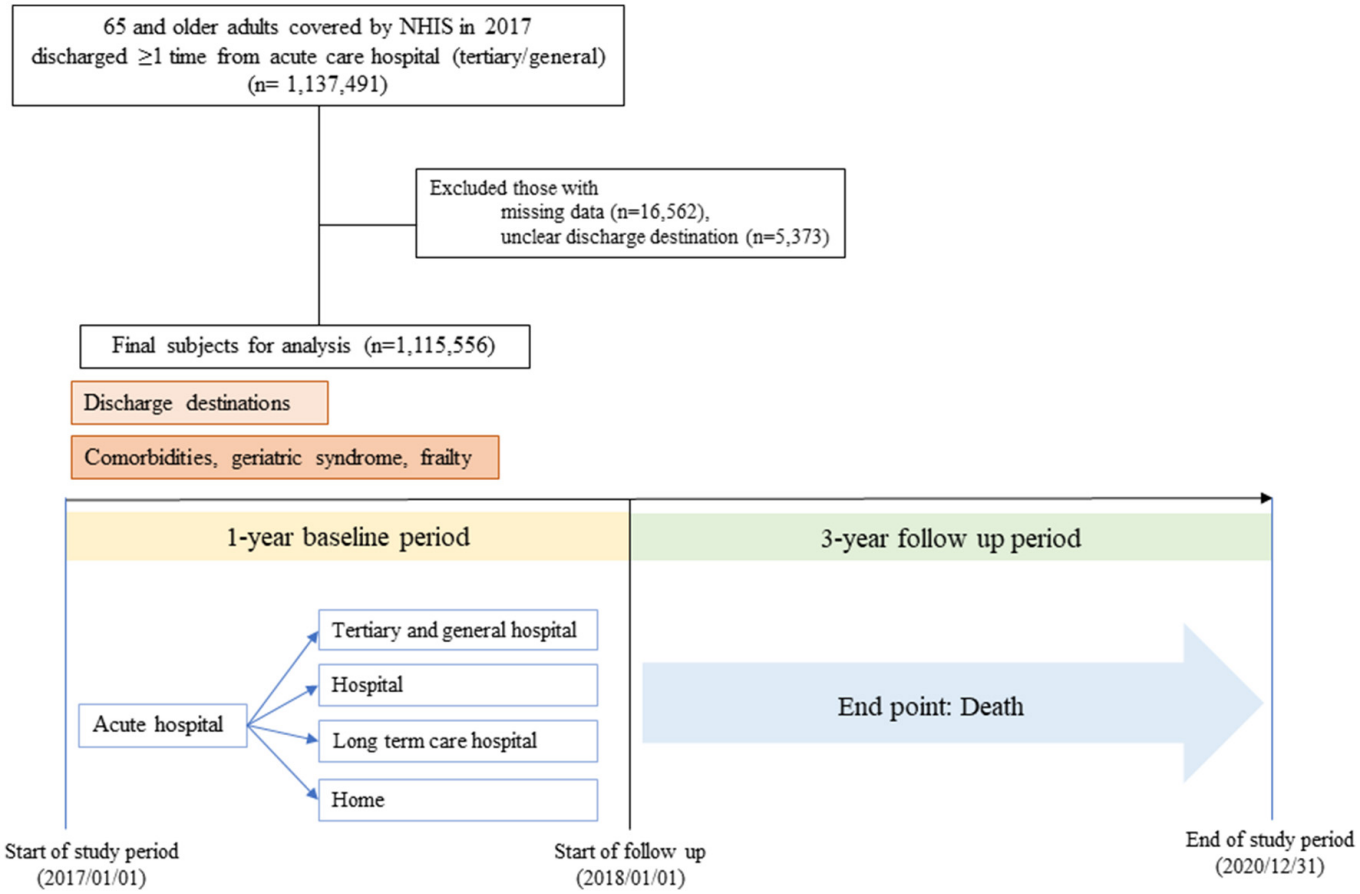
Flow chart.

The study included 1,115,556 participants with a mean age of 75.5 years (SD = 7.13). Among them, 52.8% were aged ≥75 years, and 45.6% were men. The most common discharge destination was home (853,041 patients; 76.5%), followed by tertiary or general hospitals (15.2%), long-term care hospitals (5.2%), hospitals (2.3%), and other facilities (0.8%) (Figure 1).

Geriatric syndromes were observed in 289,450 participants (25.9%), with osteoporosis-related fractures (104,315; 9.4%) and urinary incontinence (170,154; 15.3%) being the most prevalent. The mean CCI score was 4.35 (SD = 2.83), and the majority of participants (731,594; 65.6%) had a CCI score of ≥3. The mean mFI was 0.11 (SD = 0.1), with 279,017 individuals (25%) classified in the severe frailty group (Table 1).

**Table 1:** Baseline characteristics.

**Variables**	Total		Alive		Died		***p-value***
	***N***	**%**	***N***	**%**	***N***	**%**	
Total	1,115,556	100.0	694,736	100.0	420,812	100.0	
**Sex**							<0.001
Men	508,504	45.6	290,340	41.8	218,162	51.8	
Women	607,052	54.4	404,396	58.2	202,650	48.2	
Age (years)	75.5	7.1	73.4	6.1	79.1	7.3	<0.001
65–74	526,230	47.2	412,244	59.3	113,986	27.1	
≥75	589,326	52.8	282,492	40.7	306,826	72.9	
**Income**							<0.001
1st (highest)	399,722	35.8	251,259	36.2	148,458	35.3	
2nd	215,706	19.3	140,621	20.2	75,084	17.8	
3rd	139,429	12.5	88,697	12.8	50,732	12.1	
4th	107,852	9.7	68,990	9.9	38,862	9.2	
5th (lowest)	252,847	22.7	145,169	20.9	107,676	25.6	
**Region**							<0.001
Seoul metropolitan area	459,144	41.2	288,302	41.5	170,837	40.6	
Metropolitan cities	203,963	18.3	129,636	18.7	74,326	17.7	
Other areas	452,449	40.6	276,798	39.8	175,649	41.7	
**Disability**							<0.001
No	864,679	77.5	564,399	81.2	300,276	71.4	
Yes	250,877	22.5	130,337	18.8	120,536	28.6	
**Discharge destination**							<0.001
Tertiary and general hospitals	169,660	15.2	80,908	11.6	88,751	21.1	
Hospital	25,564	2.3	13,676	2.0	11,888	2.8	
Long-term care hospital	57,859	5.2	12,900	1.9	44,958	10.7	
Home	853,041	76.5	580,377	83.5	272,658	64.8	
Others	9,432	0.8	6,875	1.0	2,557	0.6	
Charlson comorbidity index	4.34800667	2.8	3.65574212	2.3	5.41773973	3.2	<0.001
0	70,122	6.3	60,049	8.6	10,073	2.4	
1–2	313,840	28.1	238,011	34.3	75,826	18.0	
≥3	731,594	65.6	396,676	57.1	334,913	79.6	
**Geriatric syndrome (number)**							<0.001
0	826,098	74.1	547,986	78.9	278,112	66.1	
1	245,996	22.1	132,391	19.1	113,605	27.0	
2	38,981	3.5	13,395	1.9	25,586	6.1	
≥3	4,473	0.4	964	0.1	3,509	0.8	
**mFI**							<0.001
Fit	143,121	12.8	100,695	14.5	42,421	10.1	
Mild frail	361,965	32.4	239,534	34.5	122,430	29.1	
Moderate frail	331,453	29.7	202,618	29.2	128,834	30.6	
Severe frail	279,017	25.0	151,889	21.9	127,127	30.2	

### 3.2 Characteristics depending on discharge destination

The mean age was highest among patients discharged to long-term care hospitals (80.70 years), followed by general hospitals (76.19 years), tertiary and general hospitals (75.80 years), and home (75.14 years) (*p* < 0.001). Regarding income level, in the highest income quintile (1st quintile), the proportion of patients discharged to tertiary/general hospitals (36.2%) and other facilities (36.3%) was higher. By contrast, among those in the lowest income quintile (5th quintile), discharge to long-term care hospitals was most common (29.8%) (*p* < 0.001).

In terms of comorbidity status, the mean CCI was highest for patients discharged to LTCHs (5.19 ± 1.23), followed by general hospitals (5.12 ± 1.15), tertiary/general hospitals (5.53 ± 1.30), and home (4.02 ± 1.05). Among patients with disabilities, 32.6% were discharged to LTCHs, which was significantly higher than for other discharge destinations (*p* < 0.001). According to discharge destinations by primary diagnosis, LTCHs had a higher proportion of patients with stroke (23.2%) and dementia (24.2%) than the other destinations. Meanwhile, patients with cancer were most frequently discharged to tertiary/general hospitals (23.4%) (*p* < 0.001).

Among patients with geriatric syndromes, discharge to LTCHs was the most common (47.8%), whereas discharge to home was the least common (23.3%). Among those with geriatric syndromes, 15.2% of patients with pressure ulcers and 5.2% of those with delirium were discharged to LTCHs (*p* < 0.001). Patients with severe frailty were most frequently discharged to general hospitals (36.7%) and least frequently discharged to home (23.3%) (*p* < 0.001; Table 2).

**Table 2:** Characteristics of discharge destinations for patients discharged from acute care hospitals.

**Variables**	Discharge destination, ***N*** (%)										***p-value***
	Tertiary/general hospitals (*n* = 169,660)		Hospital (*n* = 25,564)		Long-term care hospital (*n* = 57,859)		Home (*n* = 853 041)		Others (*n* = 9,432)		
	***N***	**%**	***N***	**%**	***N***	**%**	***N***	**%**	***N***	**%**	
**Death**											<0.001
No	80,908	47.7	13,676	53.5	12,900	22.3	580,377	68	6,875	72.9	
Yes	88,751	52.3	11,888	46.5	44,958	77.7	272,658	32	2,557	27.1	
**Sex**											
Men	84,947	50.1	10,331	40.4	20,533	35.5	389,526	45.7	3,167	33.6	
Women	84,713	49.9	15,233	59.6	37,326	64.5	463,515	54.3	6,265	66.4	
Age (years)	75.8	7.0	76.2	7.2	80.7	7.3	75.1	7.0	74.1	6.6	<0.001
65–74	76,560	45.1	10,876	42.5	11,465	19.8	422,171	49.5	5,158	54.7	
≥75	93,100	54.9	14,688	57.5	46,394	80.2	430,870	50.5	4,274	45.3	
**Income**											<0.001
1st (highest)	61,460	36.2	8,627	33.7	19,709	34.1	306,505	35.9	3,421	36.3	
2nd	32,616	19.2	4,665	18.2	9,194	15.9	167,300	19.6	1,931	20.5	
3rd	21,022	12.4	3,099	12.1	6,565	11.3	107,423	12.6	1,320	14	
4th	16,285	9.6	2,368	9.3	5,170	8.9	83,014	9.7	1,015	10.8	
5th (lowest)	38,277	22.6	6,805	26.6	17,221	29.8	188,799	22.1	1,745	18.5	
**Region**											<0.001
Seoul metropolitan area	72,073	42.5	7,423	29	20,509	35.4	355,576	41.7	3,563	37.8	
Metropolitan cities	27,991	16.5	5,043	19.7	13,110	22.7	156,288	18.3	1,531	16.2	
Other areas	69,596	41	13,098	51.2	24,240	41.9	341,177	40	4,338	46	
**Disability**											<0.001
No	128,554	75.8	18,225	71.3	39,010	67.4	671,419	78.7	7,471	79.2	
Yes	41,106	24.2	7,339	28.7	18,849	**32.6**	181,622	21.3	1,961	20.8	
Charlson comorbidity index	5.5	3.3	5.1	3.1	5.2	2.8	4.0	2.6	4.1	2.7	<0.001
0	5,345	3.2	946	3.7	763	1.3	62,406	7.3	662	7	
1–2	30,793	18.1	5,182	20.3	9,931	17.2	265,010	31.1	2,924	31	
≥3	133,522	78.7	19,436	76	47,165	81.5	525,625	61.6	5,846	62	
**Geriatric syndrome (number)**											<0.001
0	119,337	70.3	15,506	60.7	30,210	52.2	654,498	76.7	6,547	69.4	
1	41,516	24.5	7,985	31.2	20,298	35.1	173,748	20.4	2,449	26	
2	7,788	4.6	1,820	7.1	6,397	11.1	22,578	2.6	398	4.2	
≥3	1,018	0.6	253	1	953	1.6	2,211	0.3	38	0.4	
**mFI**											<0.001
Fit	16,278	9.6	1,686	6.6	4,826	8.3	119,416	14	915	9.7	
Mild frail	47,440	28	6,505	25.4	19,588	33.9	285,703	33.5	2,729	28.9	
Moderate frail	51,330	30.3	7,997	31.3	19,825	34.3	249,467	29.2	2,834	30	
Severe frail	54,612	32.2	9,376	36.7	13,620	23.5	198,455	23.3	2,954	31.3	

Bold value indicates the highest percentage within the Disability category across discharge destinations.

### 3.3 Effects of the destination of discharge on mortality

The risk of mortality was significantly higher among those discharged to tertiary/general hospitals (HR: 2.071; 95% CI: 2.055–2.087) and LTCHs (HR: 4.274; 95% CI: 4.231–4.317) compared to patients discharged home. After adjusting for age, sex, income, region, and disability status, the adjusted hazard ratios (aHRs) for mortality remained elevated for those discharged to tertiary/general hospitals (aHR: 1.977; 95% CI: 1.962–1.992), hospitals (aHR: 1.65; 95% CI: 1.619–1.68), and LTCHs (aHR: 3.492; 95% CI: 3.457–3.528). After comorbidities, geriatric syndromes, and frailty were adjusted, the aHR for mortality among patients discharged to LTCHs remained significantly higher (aHR: 2.922; 95% CI: 2.892–2.952) compared to those discharged home (Table 3).

**Table 3:** Cox regression analysis of factors related to morality.

**Variables**	**crude HR**	aHR		
	**Model 1**	**Model 2**	**Model 3**	**Model 4**
**Discharge destination**				
Tertiary and General Hospitals	2.071 (2.055–2.087)	1.977 (1.962–1.992)		1.806 (1.793–1.82)
Hospital	1.698 (1.667–1.729)	1.65 (1.619–1.68)		1.48 (1.453–1.507)
Long-term Care Hospital	4.274 (4.231–4.317)	3.492 (3.457–3.528)		2.922 (2.892–2.952)
Home	REF	REF		REF
Others	0.836 (0.804–0.87)	0.937 (0.901–0.974)		0.922 (0.887–0.959)
**Charlson comorbidity index**				
0	REF	REF	REF	REF
1–2	1.776 (1.739–1.813)	1.612 (1.579–1.646)	1.561 (1.529–1.593)	1.63 (1.596–1.665)
≥3	4 (3.922–4.08)	3.246 (3.182–3.311)	2.912 (2.855–2.971)	3.116 (3.054–3.18)
**Geriatric syndrome (number)**				
0	REF	REF	REF	REF
1	1.515 (1.505–1.526)	1.41 (1.401–1.42)	1.314 (1.305–1.323)	1.299 (1.29–1.309)
2	2.649 (2.615–2.683)	2.263 (2.234–2.293)	1.867 (1.843–1.892)	1.833 (1.809–1.857)
≥3	3.724 (3.602–3.85)	2.96 (2.863–3.061)	2.236 (2.163–2.312)	2.189 (2.117–2.263)
**mFI**				
Fit	REF	REF	REF	REF
Mild frail	1.159 (1.147–1.172)	1.03 (1.019–1.042)	0.987 (0.976–0.998)	0.851 (0.842–0.861)
Moderate frail	1.364 (1.349–1.379)	1.094 (1.082–1.106)	1.024 (1.013–1.035)	0.791 (0.782–0.8)
Severe frail	1.648 (1.63–1.667)	1.185 (1.172–1.199)	1.093 (1.081–1.105)	0.757 (0.749–0.766)

Model 1: unadjusted.

Model 2: adjusted for age, sex, income, region, disability.

Model 3: model 2 + discharge destination.

Model 4: model 3 + CCI, GS, mFI.

HR, hazard ratio; aHR, adjusted hazard ratio.

Mortality risk increased progressively with higher CCI scores, a greater number of geriatric syndromes, and more severe levels of frailty. These trends persisted even after adjusting for age, sex, income, region, disability status, and discharge destination. The aHR were 2.912 (95% CI: 2.855–2.971) for patients with a CCI score ≥3, 2.236 (95% CI: 2.163–2.312) for those with three or more geriatric syndromes, and 1.093 (95% CI: 1.081–1.105) for patients with severe frailty (Table 3). When CCI, geriatric syndromes, and the multimorbidity frailty index (mFI) were included simultaneously in the fully adjusted model (Model 4), the hazard ratios associated with frailty were attenuated and, in some categories, reversed in direction. This pattern likely reflects statistical overadjustment arising from substantial conceptual and empirical overlap among these vulnerability indicators. Because the mFI is constructed as a deficit accumulation index incorporating multimorbidity and functional impairments, adjustment for both CCI and geriatric syndromes may partition shared variance across correlated constructs, thereby altering the apparent independent association of frailty with mortality. The impact of comorbidities, geriatric syndromes, and frailty on mortality risk according to discharge destination varied when considered individually and in combination, even after adjusting for multiple confounding variables (Supplementary Table 2).

### 3.4 Impact of geriatric health state on mortality according to discharge destination

CCI was consistently associated with increased mortality risk across all discharge destinations, with the greatest risk observed among individuals with CCI scores of ≥3. This trend persisted even in Model 2, which was adjusted for age, sex, income, region, and disability. Specifically, the aHR for mortality among those with CCI ≥ 3 was 4.146 (95% CI: 3.886–4.423) for patients discharged to tertiary/general hospitals, 3.041 (95% CI: 2.616–3.535) for those discharged to hospitals, 2.341 (95% CI: 2.104–2.605) for those discharged to LTCHs, and 2.752 (95% CI: 2.693–2.812) for those discharged to home.

Similarly, the risk of mortality increased progressively with the number of geriatric syndromes, regardless of the discharge destination. Among patients with three or more geriatric syndromes, the highest risk was observed in those who were discharged home (aHR, 3.186; 95% CI: 3.035–3.344). The association of frailty with mortality risk varied according to the discharge destination. Among patients discharged, those in the most severe frailty group had a significantly increased risk of mortality (aHR, 1.261; 95% CI, 1.244–1.279) (Table 4).

**Table 4:** Cox regression analysis of factors related to morality.

**Variables**	Discharge destination, HR (95% CI)					
		**Tertiary and general hospitals**	**Hospital**	**Long-term Care Hospital**	**Home**	**Others**
Model 1	**Charlson comorbidity index**					
	0	REF	REF	REF	REF	REF
	1-2	1.965 (1.837–2.102)	1.631 (1.393–1.91)	1.989 (1.784–2.217)	1.645 (1.608–1.683)	1.494 (1.162–1.922)
	≥3	4.673 (4.38–4.985)	3.808 (3.277–4.425)	2.681 (2.41–2.983)	3.416 (3.343–3.49)	3.891 (3.067–4.938)
	**Geriatric syndrome (number)**					
	0	REF	REF	REF	REF	REF
	1	1.273 (1.254–1.293)	1.301 (1.251–1.353)	1.092 (1.07–1.114)	1.481 (1.468–1.494)	1.366 (1.253–1.488)
	2	1.814 (1.765–1.865)	1.962 (1.845–2.087)	1.24 (1.204–1.278)	2.666 (2.619–2.713)	2.102 (1.798–2.457)
	≥3	2.153 (2.009–2.308)	2.74 (2.377–3.157)	1.31 (1.222–1.405)	4.125 (3.93–4.33)	4.694 (3.208–6.869)
	**Frailty (mFI)**					
	Fit	REF	REF	REF	REF	REF
	Mild frail	0.915 (0.892–0.938)	1.046 (0.961–1.137)	0.941 (0.908–0.976)	1.152 (1.137–1.168)	0.987 (0.844–1.154)
	Moderate frail	0.908 (0.886–0.931)	1.086 (1–1.179)	0.921 (0.889–0.954)	1.403 (1.384–1.422)	1.055 (0.904–1.231)
	Severe frail	0.957 (0.935–0.981)	1.28 (1.181–1.387)	0.884 (0.851–0.917)	1.791 (1.767–1.815)	1.514 (1.304–1.757)
Model 2	**Charlson comorbidity index**					
	0	REF	REF	REF	REF	REF
	1-2	1.858 (1.737–1.987)	1.462 (1.248–1.711)	1.803 (1.618–2.011)	1.49 (1.457–1.525)	1.382 (1.074–1.778)
	≥3	4.146 (3.886–4.423)	3.041 (2.616–3.535)	2.341 (2.104–2.605)	2.752 (2.693–2.812)	3.154 (2.484–4.005)
	**Geriatric syndrome (number)**					
	0	REF	REF	REF	REF	REF
	1	1.241 (1.222–1.26)	1.256 (1.207–1.307)	1.105 (1.083–1.128)	1.374 (1.362–1.386)	1.352 (1.239–1.475)
	2	1.706 (1.659–1.754)	1.792 (1.683–1.908)	1.256 (1.219–1.294)	2.26 (2.22–2.301)	1.947 (1.663–2.28)
	≥3	1.999 (1.865–2.143)	2.371 (2.056–2.735)	1.296 (1.208–1.389)	3.186 (3.035–3.344)	3.188 (2.175–4.674)
	**Frailty (mFI)**					
	Fit	REF	REF	REF	REF	REF
	Mild frail	0.849 (0.829–0.871)	0.977 (0.898–1.063)	0.901 (0.869–0.934)	1.019 (1.006–1.033)	0.928 (0.793–1.085)
	Moderate frail	0.79 (0.771–0.81)	0.924 (0.85–1.003)	0.853 (0.823–0.884)	1.111 (1.096–1.126)	0.884 (0.756–1.033)
	Severe frail	0.768 (0.75–0.787)	0.976 (0.9–1.059)	0.794 (0.765–0.824)	1.261 (1.244–1.279)	1.079 (0.927–1.256)

Model 1: unadjusted .

Model 2: adjusted for age, sex, income, region, disability.

HR, hazard ratio; CI, confidence interval.

### 3.5 Utilization of emergency, outpatient (OPD), and inpatient services varies according to health status indicators and type of healthcare institution

The utilization of emergency, OPD, and inpatient services varies across different types of healthcare institutions, including general hospitals, long-term care hospitals, home care, and other facilities. OPD services at general hospitals were the most frequently utilized, with a mean of 40.3 outpatient visits per participant (SD = 37.5) during the follow-up period. Among patients with a CCI score of 0, OPD service usage at general hospitals averaged 26.9 visits (SD = 23.9), which was lower than those with a CCI score ≥ 3, who averaged 43.9 visits (SD = 40.7). A higher CCI score (≥3) was associated with increased utilization of inpatient services in long-term care hospitals, averaging 97.5 visits (SD = 121.6). For patients without geriatric syndromes, OPD service usage at general hospitals averaged 40.1 visits (SD = 36.4). However, this decreased to an average of 28.6 visits (SD = 35.2) for patients with three or more geriatric syndromes. Conversely, inpatient service utilization in long-term care hospitals increased significantly with the number of geriatric syndromes, reaching an average of 160.5 visits (SD = 130.0) for those with three or more syndromes. Similarly, patients with low mFI scores demonstrated an average of 23.1 OPD visits (SD = 20.7) at general hospitals, whereas those in the highest quartile (4Q) of mFI scores exhibited a marked increase, averaging 59.8 visits (SD = 48.1). Inpatient service utilization in long-term care hospitals was highest among patients in the 4Q mFI group, with an average of 90.3 visits (SD = 112.6). These findings highlight the influence of comorbidities, frailty, and geriatric syndromes on healthcare service utilization patterns across healthcare settings (Supplementary Table 3).

## 4 Discussion

### 4.1 Key findings

This nationwide, population-based cohort study analyzed over 1 million older adults discharged from acute care hospitals in South Korea, providing robust evidence of the relationships among discharge destination, comorbidities, geriatric syndromes, frailty, and mortality risk.

Discharge destination was a significant predictor of mortality. Patients discharged to LTCHs exhibited an aHR of 2.922 (95% CI: 2.892–2.952) compared to those discharged home, indicating nearly a threefold higher risk of mortality. Additionally, the presence of multiple comorbidities (CCI ≥ 3; aHR 2.912, 95% CI: 2.855–2.971), geriatric syndromes (≥3; aHR 2.236, 95% CI: 2.163–2.312), and severe frailty (aHR 1.09, 95% CI: 1.081–1.105) was independently associated with increased mortality, regardless of discharge setting. Among patients with severe frailty (aHR 1.26, 95% CI 1.244–1.279) or multiple geriatric syndromes (aHR 3.186, 95% CI 3.035–3.344), those discharged exhibited the highest relative mortality risk (aHR: 1.977; 95% CI: 1.962–1.992). This highlights the substantial vulnerability of patients with multiple comorbidities, geriatric syndrome, and frailty.

Among patients classified under “Others” as discharge destinations, those with three or more geriatric syndromes (aHR 3.188; 95% CI: 2.484–4.005) and those with severe frailty (a HR 1.079; 95% CI: 0.927–1.256) exhibited a higher mortality risk. However, the relatively wide confidence interval suggests some uncertainty regarding the magnitude of this effect. While greater frailty severity was generally associated with higher mortality risk, an unexpected trend was observed after adjusting for multiple confounding factors (adjusted for CCI and geriatric syndrome), the mortality risk appeared to decrease in patients with moderate to severe frailty (severe frailty: aHR 0.757). This finding suggests a complex interplay between frailty and mortality, warranting further investigation into the underlying mechanisms of this relationship.

Furthermore, higher levels of comorbidities and frailty were associated with a substantial increase in inpatient healthcare utilization in LTCHs. This highlights the complex interplay between health status and care environment in shaping outcomes for older adults.

### 4.2 Comparison with existing literature

Our findings align with those of previous studies, confirming that comorbidities, geriatric syndromes, and frailty contribute to increased mortality. Various studies have highlighted the significant impact of comorbidities, geriatric syndromes, and frailty on mortality among older adults. A cohort study involving 18,322 patients with heart failure demonstrated that comorbidities substantially increased mortality risk, with HR ranging from 1.16 1.93 (30). Similarly, a meta-analysis has revealed that individuals with two or more chronic conditions faced a 1.73-fold higher risk of death, which increased by 2.72-fold for those with three or more conditions (31). In Mexico, cognitive impairment and dependency have been identified as independent predictors of in-hospital mortality among older adults (32). Findings from Taiwan showed that the accumulation of geriatric syndromes significantly amplified the mortality risk (33). Furthermore, the FRADEA cohort study in Spain (34) and the SHARE study across Europe confirmed a strong association between frailty and all-cause mortality, emphasizing the critical role of disability management in those aged ≥80 years (35). These findings underscore the importance of addressing comorbidities, geriatric syndromes, and frailty in geriatric care to mitigate mortality risks.

Although existing studies have compared the impacts of comorbidities, geriatric syndromes, and frailty on mortality, they have typically been limited to specific disease groups or conducted on small populations within certain regions. By contrast, this study used big data to analyze a larger sample size, overcoming the limitations of previous small-scale cohort studies and region-specific research.

Previous studies have also reported that the discharge destination of older patients influences both mortality and healthcare utilization (7, 9, 36). In particular, the finding that transfers to long-term care hospitals or facilities are associated with higher mortality rates than discharges to home is consistent with the results of the present study. Furthermore, it distinguishes itself by comparing the mortality risks based on discharge destinations, such as institutional care settings vs. home discharge, in relation to comorbidities and geriatric syndromes, including frailty. Our study extended these results by incorporating a broader range of discharge destinations and quantifying the additive effects of frailty and geriatric syndromes across these settings.

Importantly, our study revealed that the mortality risk for vulnerable patients (i.e., those with severe frailty or multiple geriatric syndromes) discharged home may be even higher than that for those discharged to institutions, a nuance less emphasized in previous literature.

In contrast to previous research, the attenuation or reversal of the association between frailty and mortality observed in the fully adjusted model represents an important methodological consideration. This finding should not be interpreted as evidence of a protective effect of frailty, but rather as a consequence of overadjustment and collinearity arising from the simultaneous adjustment for the Charlson Comorbidity Index (CCI), geriatric syndromes, and the multimorbidity frailty index (mFI). The mFI is an ICD-10–based deficit accumulation index that incorporates multiple diseases and functional impairments, many of which conceptually overlap with CCI and geriatric syndrome variables (24). When these correlated vulnerability indicators are mutually adjusted, the shared construct of vulnerability may be statistically decomposed, leading to unstable or counterintuitive effect estimates, a phenomenon that has also been reported in prior studies using administrative data–based frailty measures (16). By contrast, in Models 1–3, which avoided redundant adjustment for overlapping vulnerability indicators, the association between frailty and mortality remained consistent with established clinical and epidemiological evidence (14). Accordingly, Model 4 should be interpreted with caution and understood as an exploratory analysis of the relative contributions of vulnerability components rather than as supporting causal inference.

Most previous studies have examined frailty, multimorbidity, geriatric syndromes, or discharge destination as independent predictors of post-discharge mortality, often within disease-specific or regionally limited cohorts (14, 31, 34). Evidence from North America and Europe consistently shows that frailty and multimorbidity are strong determinants of mortality risk after hospitalization, while other studies have emphasized elevated mortality among patients discharged to institutional care settings such as long-term care or skilled nursing facilities (6, 16, 35, 37). However, few investigations have simultaneously evaluated these interrelated domains within a unified analytical framework, despite their substantial conceptual and clinical overlap.

By integrating discharge destination, comorbidity burden, geriatric syndromes, and frailty in a single nationwide cohort of more than 1.1 million older adults, the present study extends prior work by demonstrating that post-discharge mortality risk is shaped by the interaction of multidimensional vulnerability factors, rather than by any single domain alone. Importantly, we show that severely frail individuals may face a markedly elevated risk of death even when discharged home, challenging the prevailing assumption that institutional discharge uniformly represents the highest-risk pathway (9, 36, 38). This finding highlights the limitation of using discharge destination alone as a proxy for patient vulnerability and underscores the need for risk-stratified transitional care strategies that incorporate comprehensive geriatric assessment—particularly frailty and geriatric syndromes—across care settings, with direct implications for clinical practice and health policy in aging societies (4, 6).

### 4.3 Possible explanations

Differences in mortality rates according to the discharge destination may be attributed to variations in patients' health status, socioeconomic factors, and access to healthcare resources.

Patients transferred to long-term care hospitals were older and had a higher prevalence of comorbidities, disabilities, and geriatric syndromes, which may have contributed to a poorer prognosis. Additionally, among patients with higher geriatric syndrome burden and elevated mFI scores, inpatient services were used more frequently than OPD services. Thus, individuals with greater frailty may be more likely to receive palliative or supportive care rather than active treatment, which may in turn increase mortality risk. By contrast, patients discharged home were generally in better health and more likely to benefit from family and social support.

Our findings enhance our understanding of healthcare utilization patterns in aging societies. Indicators related to frailty were strongly associated with an increased mortality risk among patients discharged home, suggesting that intensive management is particularly necessary for individuals with frailty receiving home-based care. These results have important implications for the development of tailored healthcare policies for older adults.

Among patients with severe frailty, those discharged home exhibited the highest adjusted risk of mortality, suggesting that home discharge does not necessarily ensure adequate post-discharge care for this high-risk group. We postulate that severely frail patients discharged home may experience increased mortality due to insufficient access to structured post-acute care, including regular medical follow-up, rehabilitation services, nursing care, and systematic monitoring (4, 6). In addition, the burden placed on family caregivers, delayed recognition of clinical deterioration, and fragmentation between acute hospital care and community-based services may further contribute to adverse outcomes (9, 36). These findings highlight a critical gap in current post-discharge care models and underscore the importance of risk-based discharge planning, strengthened transitional care programs, and integrated home-based medical care for severely frail older adults from both public health and policy perspectives (4, 38).

### 4.4 Clinical implications

Building on the observation that severely frail patients discharged home face substantially elevated mortality risk, these findings have important clinical and public health implications for post-discharge care delivery in older adults.

Older patients require post-acute healthcare services after hospitalization because of illness or injury (38). These services play critical roles in promoting recovery, enhancing functional capacity, and managing chronic diseases. Given the complexity and heterogeneity of healthcare needs, a disease-centered approach is insufficient. Therefore, comprehensive and integrated care strategies are required.

Our findings have significant implications for discharge planning and post-acute care management in rapidly aging societies. Discharge planning for older patients should be tailored to comorbidities, geriatric syndromes, frailty status, and intended discharge destination. Tools such as the FI and geriatric syndrome indicators facilitate the early identification of high-risk patients, offering evidence-based guidance for selecting appropriate discharge settings. This data-driven approach provides critical insights for policymakers and healthcare providers by promoting the effective integration of community-based healthcare services and resources.

For older patients discharged home, the increased risk of mortality underscores the importance of robust discharge planning and personalized post-acute care strategies. Comprehensive care programs, including home-nursing visits and rehabilitation services, should be implemented and customized to address individual patient needs. Proactive identification of high-risk patients using frailty and geriatric syndrome indices combined with enhanced home-based medical and social support can mitigate adverse outcomes.

Effective discharge planning and post-acute care for older patients require a personalized approach that incorporates assessments of frailty and geriatric syndromes. By tailoring care plans to each patient's specific health status, health outcomes could be optimized, and patient-centered continuity of care could be supported. In addition, leveraging integrated community-based healthcare resources could promote the sustainable and efficient management of healthcare needs in this population. Such individualized strategies may also reduce unnecessary hospitalizations and overuse of healthcare resources, ultimately improving the quality and continuity of care for older adults. Policymakers and clinicians should prioritize the integration of structured follow-up and community-based resources for vulnerable older adults, regardless of their discharge destination, to enhance their survival rates and improve their quality of life.

### 4.5 Limitations and strengths

This study had some limitations. First, this study utilized claims data from the National Health Insurance Service (NHIS), which lacks critical clinical information such as disease severity, functional status changes, and cognitive function. Consequently, the potential for classification errors and unmeasured confounding variables remains. Although the modified frailty index (mFI) employed in this research has been validated in prior studies, it fails to fully capture the multidimensional functional frailty states identified through comprehensive geriatric assessments. Moreover, the administrative-data-based definitions of geriatric syndromes exclude multifactorial conditions like cognitive impairment, functional decline, sensory deficits, and malnutrition, potentially underestimating the actual prevalence and clinical significance of frailty and geriatric syndromes.

Second, the operational definition of frailty and geriatric syndromes in this study relies on a simplistic summative approach, omitting key elements such as severity, duration, and interactions. This limitation poses challenges to the interpretability of the findings. As an observational study, it inherently cannot establish causal relationships. While adjustments were made for various confounders to analyze mortality risks by discharge destination, the possibility that discharge destinations reflect patients' baseline conditions cannot be entirely excluded. Additionally, critical factors such as social support, patient preferences, and regional healthcare policies, which are essential for understanding the relationship between discharge destinations and patient outcomes, were absent from the dataset.

Third, as the study is based on Korean NHIS data, its findings are contextually limited to the Korean healthcare system. The unique structural characteristics of Korea's healthcare system may hinder the direct applicability of the results to other countries. For instance, while Korean long-term care hospitals share similarities with skilled nursing facilities (SNFs) in the United States or Kaigo Iryo-in in Japan, institutional differences exist. Such discrepancies necessitate caution when generalizing the findings to other healthcare environments.

Fourth, the classification of discharge destinations in this study reflects national characteristics, potentially introducing temporal variations and selection bias. Furthermore, the classification system lacked granularity, failing to differentiate between critical categories such as skilled nursing facilities, assisted living facilities, and rehabilitation hospitals. This limitation may restrict the interpretability and generalizability of the results.

Fifth, the absence of *post-hoc* analyses precluded a more detailed exploration of outcomes in specific subgroups or the evaluation of the impact of potential confounders. Additionally, despite excluding 16,562 cases due to missing data, the study did not provide a detailed explanation of this exclusion nor conduct sensitivity analyses to assess its impact on the results. These omissions represent significant limitations, potentially undermining the reliability and generalizability of the findings.

Nevertheless, the strengths of this study include its large, nationally representative sample; comprehensive assessment of comorbidities, geriatric syndromes, and frailty; and robust follow-up for mortality using the national registry. The use of validated indices and stratified analysis by discharge destination provided nuanced insights into risk stratification.

### 4.6 Future directions

Future research should prioritize the standardization of quantitative assessment tools for frailty and geriatric syndromes to enhance the reliability and reproducibility of analyses. This approach will enable large-scale studies that include diverse populations, thereby increasing the generalizability of findings. Additionally, to elucidate the relationships between discharge destinations, geriatric health conditions, and mortality, prospective studies integrating detailed clinical and functional data, as well as the perspectives of patients and caregivers, are essential. Such studies can leverage advanced methodologies, including clustering techniques and machine learning approaches, to provide a deeper understanding of the complex interactions between these factors. Particular emphasis should be placed on conducting interventional studies aimed at evaluating the effectiveness of personalized transitional care programs for frail older adults. These programs are expected to play a significant role in improving post-discharge health outcomes, reducing hospital readmissions, and lowering mortality rates among this vulnerable population. Furthermore, research should focus on identifying the impact of modifiable factors, such as healthcare quality, resource availability, staffing patterns, and discharge environments, on mortality. Investigating these factors will provide critical insights for developing refined strategies to reduce mortality and improve health outcomes in the growing older population. Such comprehensive and multi-faceted research efforts will contribute to advancing clinical practices and healthcare policies, ultimately enhancing the quality of care and outcomes for older adults.

## 5 Conclusions

Discharge destination, comorbidities, geriatric syndrome, and frailty independently and interactively exerted a significant impact on post-hospitalization mortality risk among older adults. Discharged older adults with severe frailty or multiple geriatric syndromes had the greatest risk of mortality. Thus, individualized post-discharge management strategies targeting this particularly vulnerable population are urgently needed. Early identification of high-risk individuals and implementation of optimized transitional care may contribute to improved survival and quality of life in aging populations.

## Data Availability

The data analyzed in this study is subject to the following licenses/restrictions: this study was performed using the National Health Insurance System database (https://nhiss.nhis.or.kr/en/z/a/001/lpza001m01en.do), and the results do not necessarily represent the opinions of the National Health Insurance Corporation. Restrictions apply to the availability of these data, which were used under the license for this study. Requests to access these datasets should be directed to National Health Insurance Corporation.
