# Supplementary material for: Influence of comorbidities, geriatric syndromes, and frailty on mortality risk by discharge destination in older adults after acute hospitalization: a nationwide cohort study
Source: Front Public Health. 2026 Feb 27;14:1754972. doi: 10.3389/fpubh.2026.1754972 (PMC12983232; doi:10.3389/fpubh.2026.1754972)
Supplement: Supplementary file 1 [file Table_1.docx]

**Supplementary Table 1-1.** Charlson Comorbidity Index (CCI) ICD-10 code

| Condition | ICD-10 Coding Algorithms | Weight |
| --- | --- | --- |
| Myocardial infarction | I21, I22, I25.2 | 1 |
| Congestive heart failure | I09.9, I11.0, I13.0, I13.2, I25.5, I42.x, I43.x, I50.x, P29.0 | 1 |
| Peripheral vascular disease | I70.x, I71.x, I73.1, I73.8, I73.9, I77.1, I79.0, I79.2, K55.1, K55.8, K55.9, Z95.8, Z95.9 | 1 |
| Cerebrovascular disease | G45.x, G46.x, H34.0, I60.x–I69.x | 1 |
| Dementia | F00.x–F03.x, F05.1, G30.x, G31.1 | 1 |
| Chronic pulmonary disease | I27.8, I27.9, J40.x–J47.x, J60.x–J67.x, J68.4, J70.1, J70.3 | 1 |
| Connective tissue disease | M05.x, M06.x, M31.5, M32.x–M34.x, M35.1, M35.3, M36.0 | 1 |
| Peptic ulcer disease | K25.x–K28.x | 1 |
| Mild liver disease | B18.x, K70.0–K70.3, K70.9, K71.3–K71.5, K71.7, K73.x, K74.x, K76.0, K76.2–K76.4, K76.8, K76.9, Z94.4 | 1 |
| Diabetes without complications | E10.0, E10.1, E10.6, E10.8, E10.9, E11.0, E11.1, E11.6, E11.8, E11.9, E12.x–E14.x | 1 |
| Hemiplegia or paraplegia | G04.1, G11.4, G80.1, G80.2, G81.x, G82.x, G83.0–G83.4, G83.9 | 2 |
| Renal disease | I12.0, I13.1, N03.2–N03.7, N05.2–N05.7, N18.x, N19.x, N25.0, Z49.0–Z49.2, Z94.0, Z99.2 | 2 |
| Diabetes with complications | E10.2–E10.5, E10.7, E11.2–E11.5, E11.7 | 2 |
| Malignancy | C00.x–C26.x, C30.x–C34.x, C37.x–C41.x, C43.x, C45.x–C58.x, C60.x–C76.x, C81.x–C85.x, C88.x, C90.x–C97.x | 2 |
| Moderate or severe liver disease | I85.0, I85.9, I86.4, I98.2, K70.4, K71.1, K72.1, K72.9, K76.5, K76.6, K76.7 | 3 |
| Metastatic solid tumor | C80.x | 6 |
| AIDS/HIV | B20.x–B24.x | 6 |

**Supplementary Table 1-2.** mFI ICD-10 code

| ICD-10-CM | Deficits (one diagnosis in the inpatient settings or at least three diagnoses in outpatient settings) |
| --- | --- |
| E87 | Disorders of electrolyte and fluid balance |
| F03 | Dementia |
| G47 | Sleep disorders and apnea |
| H02 | Disorders of eyelids |
| H35 | Retinopathy and other eye disorders |
| H40 | Glaucoma and ocular hypertension |
| H81 | Vertigo or other disorder of vestibular function |
| I10 | Hypertension |
| I11 | Hypertensive heart disease with/without heart failure |
| I20 | Angina |
| I25 | Atherosclerotic heart disease and chronic ischemic heart disease |
| I48 | Atrial fibrillation and atrial flutter |
| I49 | Cardiac arrhythmia |
| I50 | Heart Failure |
| I63 | Cerebral infarction |
| I67 | Cerebral vascular disease |
| I69 | Late effect of cerebrovascular diseases |
| J18 | Pneumonia |
| J44 | Chronic obstructive pulmonary disease |
| J45 | Asthma |
| K25 | Gastric ulcer |
| K27 | Peptic ulcer |
| K30 | Functional dyspepsia |
| K59 | Constipation |
| L03 | Cellulitis |
| L30 | Dermatitis |
| M10 | Gout |
| M15 | Polyosteoarthritis |
| M19 | Osteoarthritis |
| M48 | Spinal stenosis and spondylopathy |
| M81 | Osteoporosis |
| N18 | Chronic kidney disease |
| N39 | Urinary tract infection |
| N40 | Enlarged and nodular prostate |
| R05 | Cough |
| R10 | Abdominal pain |
| R42 | Dizziness and giddiness |
| Z96 | Presence of functional implant |

**Supplementary Table 2.**

| Variables | Model 1 | Model 2 | Model 3 | Model 4 | Model 5 | Model 6 | Model 7 | Model 8 | Model 9 |
| --- | --- | --- | --- | --- | --- | --- | --- | --- | --- |
| **Discharge Destination** |  |  |  |  |  |  |  |  |  |
| Tertiary and General Hospitals | 2.071 (2.055-2.087) | 1.977 (1.962-1.992) | 1.827 (1.813-1.841) | 1.924 (1.909-1.939) | 1.956 (1.941-1.971) | 1.785 (1.772-1.799) | 1.843 (1.829-1.857) | 1.916 (1.902-1.931) | 1.806 (1.793-1.82) |
| Hospital | 1.698 (1.667-1.729) | 1.65 (1.619-1.68) | 1.537 (1.509-1.566) | 1.55 (1.521-1.579) | 1.628 (1.599-1.659) | 1.456 (1.429-1.483) | 1.559 (1.53-1.588) | 1.543 (1.514-1.571) | 1.48 (1.453-1.507) |
| Long-term Care Hospital | 4.274 (4.231-4.317) | 3.492 (3.457-3.528) | 3.19 (3.157-3.222) | 3.18 (3.147-3.213) | 3.499 (3.464-3.535) | 2.945 (2.915-2.976) | 3.185 (3.153-3.218) | 3.189 (3.156-3.222) | 2.922 (2.892-2.952) |
| Home | REF | REF | REF | REF | REF | REF | REF | REF | REF |
| Others | 0.836 (0.804-0.87) | 0.937 (0.901-0.974) | 0.929 (0.893-0.966) | 0.911 (0.876-0.947) | 0.927 (0.891-0.964) | 0.906 (0.872-0.942) | 0.941 (0.905-0.979) | 0.908 (0.873-0.944) | 0.922 (0.887-0.959) |
| **Charlson Comorbidity Index** | |  |  |  |  |  |  |  |  |
| 0 | REF | REF | REF |  |  | REF | REF |  | REF |
| 1-2 | 1.776 (1.739-1.813) | 1.612 (1.579-1.646) | 1.561 (1.529-1.593) |  |  | 1.551 (1.519-1.584) | 1.625 (1.592-1.66) |  | 1.63 (1.596-1.665) |
| ≥3 | 4 (3.922-4.08) | 3.246 (3.182-3.311) | 2.912 (2.855-2.971) |  |  | 2.851 (2.794-2.908) | 3.121 (3.058-3.184) |  | 3.116 (3.054-3.18) |
| **Geriatric syndrome (number)** | |  |  |  |  |  |  |  |  |
| 0 | REF | REF |  | REF |  | REF |  | REF | REF |
| 1 | 1.515 (1.505-1.526) | 1.41 (1.401-1.42) |  | 1.314 (1.305-1.323) |  | 1.276 (1.267-1.285) |  | 1.308 (1.299-1.318) | 1.299 (1.29-1.309) |
| 2 | 2.649 (2.615-2.683) | 2.263 (2.234-2.293) |  | 1.867 (1.843-1.892) |  | 1.787 (1.764-1.811) |  | 1.853 (1.829-1.878) | 1.833 (1.809-1.857) |
| ≥3 | 3.724 (3.602-3.85) | 2.96 (2.863-3.061) |  | 2.236 (2.163-2.312) |  | 2.132 (2.062-2.204) |  | 2.218 (2.145-2.294) | 2.189 (2.117-2.263) |
| **Frailty (mFI)** |  |  |  |  |  |  |  |  |  |
| Fit | REF | REF |  |  | REF |  | REF | REF | REF |
| Mild frail | 1.159 (1.147-1.172) | 1.03 (1.019-1.042) |  |  | 0.987 (0.976-0.998) |  | 0.863 (0.854-0.873) | 0.973 (0.962-0.984) | 0.851 (0.842-0.861) |
| Moderate frail | 1.364 (1.349-1.379) | 1.094 (1.082-1.106) |  |  | 1.024 (1.013-1.035) |  | 0.818 (0.809-0.827) | 0.991 (0.98-1.002) | 0.791 (0.782-0.8) |
| Severe frail | 1.648 (1.63-1.667) | 1.185 (1.172-1.199) |  |  | 1.093 (1.081-1.105) |  | 0.807 (0.798-0.817) | 1.025 (1.014-1.037) | 0.757 (0.749-0.766) |

Model 1 unadjusted

Model 2 adjusted for age, sex, income, regions, disability

Model 3 model 2+ CCI

Model 4 model 2+ GS

Model 5 model 2+ mFI

Model 6 model 2+ CCI, GS

Model 7 model 2+ CCI, mFI

Model 8 model 2+ GS, mFI

Model 9 model 2+ CCI, GS, mFI

**Supplementary Table 3.** Healthcare utilization

|  | Total | | | | | | Tertiary and general hospitals | | | | | | Hospital | | | | | | | Long-term care hospital | | | | | | | | Home | | | | | | | | Others | | | | | | | |
| --- | --- | --- | --- | --- | --- | --- | --- | --- | --- | --- | --- | --- | --- | --- | --- | --- | --- | --- | --- | --- | --- | --- | --- | --- | --- | --- | --- | --- | --- | --- | --- | --- | --- | --- | --- | --- | --- | --- | --- | --- | --- | --- | --- |
|  | ER | | OPD | | Admission | | ER | | OPD | | Admission | | ER | | OPD | | Admission | | | ER | | | OPD | | Admission | | | ER | | | OPD | | Admission | | | ER | | | OPD | | Admission | | |
|  | Mean | SD | Mean | SD | Mean | SD | Mean | SD | Mean | SD | Mean | SD | Mean | SD | Mean | SD | Mean | SD | Mean | | SD | Mean | | SD | Mean | SD | Mean | | SD | Mean | | SD | Mean | SD | Mean | | SD | Mean | | SD | Mean | SD |  |
| Total | 6.0 | 15.7 | 40.3 | 37.5 | 35.4 | 72.4 | 10.4 | 22.5 | 39.1 | 39.6 | 47.8 | 77.2 | 5.3 | 17.3 | 37.7 | 41.8 | 86.8 | 117.1 | 5.2 | | 20.5 | 12.3 | | 25.0 | 189.4 | 148.4 | 5.2 | | 13.3 | 42.4 | | 36.7 | 20.9 | 42.3 | 3.4 | | 14.3 | 51.7 | | 44.5 | 35.4 | 63.7 |  |
| **Charlson comorbidity index** | | | | | | |  |  |  |  |  |  |  |  |  |  |  |  |  | |  |  | |  |  |  |  | |  |  | |  |  |  |  | |  |  | |  |  |  |  |
| 0 | 2.5 | 8.1 | 26.9 | 23.9 | 11.8 | 30.2 | 3.7 | 15.1 | 27.7 | 24.3 | 15.1 | 38.0 | 2.0 | 10.1 | 30.5 | 28.6 | 47.3 | 92.1 | 1.4 | | 7.2 | 20.9 | | 31.7 | 119.8 | 136.0 | 2.5 | | 7.1 | 26.7 | | 23.5 | 9.5 | 19.2 | 1.0 | | 4.9 | 36.6 | | 32.1 | 18.5 | 30.1 |  |
| 1–2 | 3.6 | 10.7 | 34.9 | 29.9 | 21.4 | 54.5 | 6.0 | 16.9 | 34.1 | 30.7 | 27.0 | 57.9 | 2.7 | 13.1 | 37.1 | 35.5 | 53.8 | 94.0 | 2.8 | | 18.0 | 11.5 | | 22.4 | 194.5 | 154.2 | 3.4 | | 9.2 | 35.7 | | 29.5 | 13.5 | 28.7 | 1.7 | | 9.4 | 45.5 | | 36.0 | 24.5 | 46.7 |  |
| ≥3 | 7.3 | 17.8 | 43.9 | 40.7 | 43.7 | 80.2 | 11.6 | 23.6 | 40.7 | 41.7 | 53.8 | 80.9 | 6.2 | 18.4 | 38.3 | 43.8 | 97.5 | 121.6 | 5.8 | | 21.1 | 12.3 | | 25.4 | 189.5 | 147.1 | 6.4 | | 15.3 | 47.6 | | 40.0 | 26.0 | 48.8 | 4.6 | | 16.7 | 56.5 | | 48.5 | 42.7 | 72.2 |  |
| **Geriatric syndrome (number)** | | | | | | |  |  |  |  |  |  |  |  |  |  |  |  |  | |  |  | |  |  |  |  | |  |  | |  |  |  |  | |  |  | |  |  |  |  |
| 0 | 4.8 | 13.3 | 40.1 | 36.4 | 27.8 | 61.9 | 8.6 | 19.6 | 39.2 | 38.6 | 39.4 | 67.0 | 4.1 | 14.7 | 38.5 | 41.3 | 78.1 | 112.9 | 4.2 | | 19.4 | 12.6 | | 25.1 | 188.8 | 151.4 | 4.2 | | 11.2 | 41.4 | | 35.7 | 17.1 | 35.0 | 2.8 | | 13.3 | 50.6 | | 42.6 | 29.5 | 56.9 |  |
| 1 | 8.1 | 19.2 | 42.0 | 40.2 | 50.4 | 87.1 | 13.2 | 26.2 | 40.1 | 41.8 | 60.9 | 88.6 | 6.3 | 18.2 | 37.9 | 42.5 | 92.4 | 119.1 | 5.6 | | 20.9 | 12.2 | | 25.1 | 189.2 | 146.5 | 7.4 | | 16.8 | 45.9 | | 39.5 | 29.9 | 53.6 | 4.3 | | 15.6 | 54.6 | | 48.5 | 44.2 | 70.7 |  |
| 2 | 14.2 | 26.7 | 36.1 | 41.1 | 90.4 | 112.3 | 20.0 | 33.0 | 34.5 | 43.1 | 95.8 | 110.1 | 10.1 | 26.2 | 32.3 | 41.9 | 126.0 | 128.2 | 7.8 | | 22.5 | 11.6 | | 25.1 | 192.5 | 141.8 | 14.4 | | 24.9 | 43.6 | | 41.1 | 57.0 | 79.1 | 6.7 | | 17.7 | 52.5 | | 48.4 | 70.7 | 91.8 |  |
| ≥3 | 21.0 | 32.5 | 28.6 | 35.2 | 124.5 | 121.1 | 25.8 | 37.9 | 29.5 | 36.4 | 125.2 | 118.3 | 16.6 | 32.6 | 24.0 | 37.7 | 160.5 | 130.0 | 11.0 | | 27.3 | 11.4 | | 20.4 | 192.1 | 137.1 | 23.7 | | 30.8 | 35.8 | | 36.7 | 91.4 | 98.6 | 17.2 | | 34.7 | 42.7 | | 38.0 | 95.2 | 118.5 |  |
| **mFI, mean (SD)** | | | | | | |  |  |  |  |  |  |  |  |  |  |  |  |  | |  |  | |  |  |  |  | |  |  | |  |  |  |  | |  |  | |  |  |  |  |
| 1Q | 3.9 | 11.4 | 23.1 | 20.7 | 21.7 | 54.4 | 7.2 | 17.5 | 22.5 | 22.5 | 33.3 | 57.1 | 2.6 | 11.9 | 20.3 | 25.0 | 66.3 | 111.4 | 2.8 | | 19.1 | 6.3 | | 14.8 | 177.1 | 153.6 | 3.5 | | 9.7 | 23.9 | | 20.2 | 13.2 | 29.1 | 2.8 | | 15.1 | 29.2 | | 27.1 | 27.1 | 56.1 |  |
| 2Q | 4.8 | 13.7 | 31.3 | 27.5 | 31.5 | 71.1 | 8.5 | 20.9 | 28.9 | 28.6 | 41.3 | 72.0 | 3.8 | 15.3 | 26.0 | 29.1 | 84.6 | 121.0 | 3.6 | | 18.2 | 8.1 | | 17.9 | 197.2 | 152.0 | 4.3 | | 11.5 | 33.4 | | 27.0 | 17.3 | 37.9 | 2.6 | | 14.7 | 38.8 | | 32.4 | 29.6 | 57.1 |  |
| 3Q | 6.1 | 15.9 | 41.1 | 35.0 | 38.2 | 76.8 | 10.3 | 22.4 | 36.9 | 35.1 | 49.1 | 80.9 | 4.7 | 15.4 | 34.4 | 36.7 | 88.8 | 119.8 | 5.2 | | 20.0 | 11.7 | | 23.0 | 192.5 | 148.1 | 5.4 | | 13.8 | 44.4 | | 34.4 | 22.1 | 44.6 | 3.0 | | 12.5 | 50.4 | | 39.2 | 36.1 | 67.0 |  |
| 4Q | 8.4 | 19.1 | 59.8 | 48.1 | 44.2 | 75.2 | 13.0 | 24.8 | 55.1 | 49.3 | 56.4 | 81.8 | 7.5 | 20.4 | 51.9 | 50.5 | 90.3 | 112.6 | 8.3 | | 24.0 | 21.5 | | 35.0 | 178.2 | 140.8 | 7.3 | | 16.5 | 64.0 | | 47.0 | 29.5 | 50.0 | 4.8 | | 15.2 | 71.9 | | 54.1 | 42.4 | 67.6 |  |

ER, emergency room; OPD, outpatient department; mFI, multimorbidity frailty index; SD, standard deviation
